# Enhanced Surgical Efficiency with 3D Heads-Up Visualization in Vitreoretinal Surgery: A Retrospective Comparative Study

**DOI:** 10.3390/jcm15093485

**Published:** 2026-05-02

**Authors:** Ludovico Iannetti, Carmen Baratta, Annalisa Romaniello, Claudia Magnolo, Francesco Ruggeri, Francesca Romana Blasi, Sandra Cinzia Carlesimo, Magda Gharbiya, Fabio Scarinci, Ludovico Alisi

**Affiliations:** 1 Policlinico Umberto I University Hospital, 00185 Rome, Italyfrancescaromana.blasi@uniroma1.it (F.R.B.); sandracinzia.carlesimo@uniroma1.it (S.C.C.); magda.gharbiya@uniroma1.it (M.G.); 2 Ospedale San Biagio, Domodossola ASL Verbano Cusio Ossola, 28845 Domodossola, Italy; carmen.baratta@uniroma1.it; 3 Department of Sense Organs, Sapienza University of Rome, 00185 Rome, Italy; annalisa.romaniello@uniroma1.it (A.R.); claudia.magnolo@uniroma1.it (C.M.); francesco.ruggeri@uniroma1.it (F.R.);; 4 Ophthalmology Unit, San Giovanni Addolorata Hospital, 00185 Rome, Italy

**Keywords:** vitreoretinal surgery, 3D heads-up visualization, pars plana vitrectomy, epiretinal membrane, macular hole, retinal detachment, surgical ergonomics

## Abstract

**Background/Objectives:** This study analyzed intraoperative parameters, structural safety, and morphofunctional outcomes of vitreoretinal procedures performed using a conventional operating microscope versus a three-dimensional (3D) heads-up digital visualization system. **Methods:** A retrospective single-surgeon case series included 248 eyes undergoing pars plana vitrectomy for epiretinal membrane (ERM), macular hole (MH), or rhegmatogenous retinal detachment (RRD). Patients were divided into conventional microscope (*n* = 122) and 3D heads-up (*n* = 126) groups. Primary outcomes included surgical duration, endoillumination intensity, best-corrected visual acuity (BCVA), anatomical success, and complications over a 6-month follow-up. **Results:** The 3D cohort showed a significantly shorter global median surgical duration (50.0 vs. 60.0 min, *p* = 0.001). Multivariate regression confirmed the 3D system as an independent predictor of shorter operative time globally (*p* = 0.011). After adjusting for baseline disease severity imbalances in the ERM subgroup, the 3D system maintained an independent reduction in surgical duration of 5.5 min (*p* = 0.044). The 3D system also required significantly lower endoillumination across all procedures (*p* ≤ 0.002). Anatomical success rates were high and comparable across indications. Both groups achieved similar and significant visual improvement at 6 months (*p* = 0.120). Structural safety biomarkers (SANFL, DONFL) and complication rates remained comparable. **Conclusions:** The 3D heads-up visualization system demonstrated comparable functional and anatomical outcomes to conventional microscopy across standard vitreoretinal procedures. It allows for surgery under significantly lower light conditions and demonstrates the potential to optimize operative time, particularly in ERM peeling. Furthermore, it maintains an equivalent structural safety profile to conventional surgery.

## 1. Introduction

The introduction of three-dimensional (3D) heads-up visualization systems has marked a significant evolution in vitreoretinal surgery. These systems have introduced a novel approach to ophthalmic microsurgery by combining digital stereoscopic imaging, enhanced depth perception, and ergonomic improvements that contribute to both surgical precision and operator well-being. Unlike conventional optical microscopes, 3D visualization platforms allow surgeons to perform procedures while viewing a high-definition stereoscopic image projected on a large digital screen, rather than through binocular eyepieces. This configuration enables the surgeon to maintain a more natural and upright posture, reducing fatigue and musculoskeletal strain during long and demanding procedures [1,2].

Other studies have highlighted additional advantages of this approach, including improved depth of field, better image resolution, and enhanced magnification, which may facilitate the identification of fine retinal structures such as membranes or vitreous remnants [3]. The digital interface of these systems also allows for integrated visualization of imaging modalities and real-time surgical parameters on the same display, potentially improving both surgical accuracy and team communication [1].

Furthermore, the ability to operate with lower endoillumination levels—thanks to increased light sensitivity and digital signal amplification—has been associated with a potentially reduced risk of retinal phototoxicity [4]. The shared 3D view also offers significant educational advantages, as all members of the surgical team can observe the same stereoscopic image, replicating the surgeon’s perception of depth, and early experiences reported a brief learning curve related to hand–eye coordination [1,2,5].

Some studies have compared 3D heads-up visualization systems with conventional operating microscopes in vitreoretinal surgery. However, few authors have included comparative cohorts of eyes operated on by a single surgeon, providing a more consistent surgical approach and allowing for a more reliable comparison between techniques [2,5,6,7,8,9].

In this study, we report a comparative analysis of vitreoretinal diseases operated on using a conventional operating microscope and the Ngenuity^®^ 3D Visualization System.

## 2. Materials and Methods

This was a retrospective, comparative, consecutive cohort study. We reviewed the medical records of consecutive patients who underwent pars plana vitrectomy (PPV) between 2021 and 2024 at Policlinico Umberto I of Rome. The study adhered to the tenets of the Declaration of Helsinki, and informed consent was obtained from all patients.

Inclusion criteria were the presence of symptomatic epiretinal membrane (ERM), full-thickness macular hole (MH), or rhegmatogenous retinal detachment (RRD) requiring surgical intervention. Exclusion criteria included previous vitreoretinal surgery, dense media opacities preventing adequate visualization, or follow-up shorter than 6 months. ERM staging was assessed via the Govetto classification [10]. MH were classified based on the canonical Gass staging system [11].

Patients were assigned to one of two groups based on the visualization system utilized: a conventional microscope group, with surgery performed using a conventional analog operating microscope, and a 3D heads-up group, with surgery performed using a high-dynamic-range (HDR) 3D digital visualization system (Ngenuity^®^ 3D Visualization System, Alcon, Fort Worth, TX, USA).

All surgeries were performed by the same experienced surgeon (L.I.) using a standard 25- or 27-gauge transconjunctival sutureless pars plana vitrectomy (PPV) system. For ERM, core vitrectomy was followed by staining with vital dyes (Brilliant Blue G or Dual Blue). The internal limiting membrane (ILM) was peeled in all cases. For MH, determination of MH stage and measurement of the minimum linear diameter (MLD) were performed preoperatively using spectral domain optical coherence tomography (SD-OCT) (Heidelberg Engineering, Heidelberg, Germany). The surgical procedure involved ILM peeling, and the “inverted flap” technique was employed for large (>400 µm) or myopic holes. For RRD, the surgical strategy included core vitrectomy, shaving of the vitreous base, fluid–air exchange, and endolaser photocoagulation around retinal breaks. Scleral buckling (SB) was combined with PPV in selected cases based on the surgeon’s judgment. For tamponade and phacoemulsification, fluid–air exchange was followed by tamponade with sterile air, 20% sulfur hexafluoride (SF6), 12–14% perfluoropropane (C3F8), or silicone oil (SO) depending on the pathology and retinal status. Combined phacoemulsification with intraocular lens (IOL) implantation was performed in phakic eyes with cataract or to prevent post-vitrectomy cataract progression. Intraoperative parameters, specifically the duration of surgery (minutes) and the endoillumination intensity (lumens), were recorded for every case.

### 2.1. Outcome Measures: Data Collected at Baseline and 1 Month, 3 Months, and 6 Months Postoperatively

Functional Outcomes: Best-corrected visual acuity (BCVA) was measured using Snellen charts and converted to logMAR for statistical analysis. Delta BCVA was calculated as the difference between preoperative and 6-month postoperative BCVA (positive values indicate visual gain).

### 2.2. Anatomical Outcomes

ERM/MH: The primary outcome was central macular thickness (CMT) assessed using SD-OCT, while in eyes with MH the primary outcome was anatomical closure. RRD: the primary outcome was retinal reattachment after a single surgery. In this latter group, secondary outcomes included the rate of RD recurrence within 3 months and macula on/off status at presentation. Complications: The incidence of postoperative cystoid macular edema (CME) was evaluated at 6 months in all groups. Structural safety biomarkers: To assess iatrogenic mechanical trauma to the retinal surface, SD-OCT scans were analyzed for the presence of swelling of the arcuate nerve fiber layer (SANFL) defects and dissociated optic nerve fiber layer (DONFL) appearance at 1, 3, and 6 months for the EMR and MH groups.

### 2.3. Statistical Analysis

Statistical analysis was performed using R Studio (2026.01.0 Build 392, Posit team (2025). RStudio: Integrated Development Environment for R. Posit Software, PBC, Boston, MA). The normality of data distribution was assessed using the Shapiro–Wilk test and visual inspection of histograms. Continuous variables (e.g., age, surgery duration, BCVA, CMT) were non-normally distributed and are reported as medians and interquartile ranges [IQRs]. Comparison between groups was performed using the Mann–Whitney U test. Categorical variables (e.g., gender, tamponade type, anatomical success, SANFL/DONFL presence) are reported as frequencies and percentages and compared using Fisher’s exact test. For global analysis, a merged dataset comprising all three indications (ERM, MH, RD) was created to analyze overall surgical efficiency and safety profiles. A linear regression model was applied to the 3D group to assess the correlation between surgical duration and the consecutive case sequence number. The Pearson correlation coefficient (r) and slope were calculated. To account for potential confounding factors, multivariate linear regression models were developed. For surgical duration, the model adjusted for age, surgical indication (ERM, MH, RD), baseline BCVA, and the performance of combined phacoemulsification. For visual outcomes (delta BCVA), the model adjusted for baseline BCVA, age, and surgical indication to correct for the ceiling/floor effect of preoperative visual acuity. A separate sub-analysis using linear regression was performed on the ERM cohort to isolate the specific impact of the 3D visualization system on operative time independently of combined cataract surgery and disease severity (ERM stage). A *p*-value < 0.05 was considered statistically significant.

## 3. Results

### 3.1. Global Cohort Analysis

A total of 248 eyes were included in the study: 122 in the conventional microscope group and 126 in the 3D heads-up group. The two groups were well balanced regarding age (median 70 vs. 72 years, *p* = 0.555) and gender (*p* = 0.800). There was no significant difference in the distribution of surgical indications (ERM, MH, RD) between the two visualization systems (*p* = 0.356). The rate of combined phacoemulsification was high and nearly identical in both groups (64.8% vs. 64.3%, *p* = 1.000). Results are summarized in Table 1.

The use of the 3D digital system was associated with a significant reduction in surgical duration. The median operative time was 50 min (IQR 40, 75) for the 3D group compared to 60 min (IQR 50, 90) for the conventional group (*p* = 0.001). This 10 min reduction in median operative time was consistent across the global cohort.

Preoperatively, the conventional group presented with significantly lower visual acuity compared to the 3D group (median 1.0 vs. 0.7 logMAR, *p* = 0.001). Postoperatively, both groups achieved significant visual improvement. At 6 months, final BCVA was comparable between groups (0.40 vs. 0.20 logMAR, *p* = 0.120).

The median visual gain (delta BCVA) was significantly higher in the conventional group (0.40 logMAR) than in the 3D group (0.30 logMAR) (*p* = 0.004). To account for potential baseline confounders, including age, combined phacoemulsification, specific pathology, and baseline visual acuity, multivariate linear regression models were performed for surgical duration and visual gain. After adjusting for confounders, the use of the 3D heads-up system remained an independent predictor of shorter surgical time. The model estimated a net reduction of 10.8 min per surgery in the 3D group compared to the conventional microscope (coefficient: −10.8 [95% CI: −19.0 to −2.46], *p* = 0.011). Regarding visual recovery (delta BCVA), the multivariate analysis revealed no significant difference between the two visualization systems (*p* = 0.442). The incidence of postoperative CME was similar between the groups (21.3% conventional vs. 17.5% 3D, *p* = 0.521), indicating no increased inflammatory risk associated with the digital interface. To address potential chronological bias, an analysis of the adoption timeline revealed an initial period of conventional surgery (2021–2022), a transitional overlap period (2023, n = 88 cases mixed), and a final period predominantly utilizing 3D surgery (2024). A sensitivity analysis restricted solely to the transitional overlap year of 2023 showed comparable median surgical durations (57.5 min conventional vs. 55.0 min 3D). Multivariate regression isolated to 2023 demonstrated no significant difference in operative time between the two platforms (*p* = 0.664).

### 3.2. ERM

A total of 122 eyes were included, of which 55 were in the conventional microscope group and 67 in the 3D heads-up group.

Baseline demographic characteristics considering age (median 75.5 vs. 75.2 years, *p* = 0.474) and gender (*p* = 0.275) were comparable between groups.

However, a statistically significant difference was observed in the preoperative ERM staging (*p* = 0.005). The conventional group presented with a higher proportion of stage 4 ERMs (69.1%) compared to the 3D group (44.8%), whereas the 3D group had a higher prevalence of stage 3 ERMs (40.3% vs. 14.5%). Preoperative BCVA and CMT were similar between the two groups (*p* = 0.132 and *p* = 0.278, respectively). The rate of combined phacoemulsification was high and similar in both groups (63.6% vs. 65.7%, *p* = 0.851) (Table 2).

#### 3.2.1. Surgical Efficiency and Intraoperative Parameters

The duration of surgery was significantly shorter in the 3D group compared to the conventional group (median: 40.0 min vs. 50.0 min; *p* = 0.012).

Furthermore, the endoillumination intensity required during peeling was substantially lower in the 3D group (median: 15 vs. 35; *p* < 0.001), highlighting the superior light sensitivity of the digital sensors.

**Figure 1:**
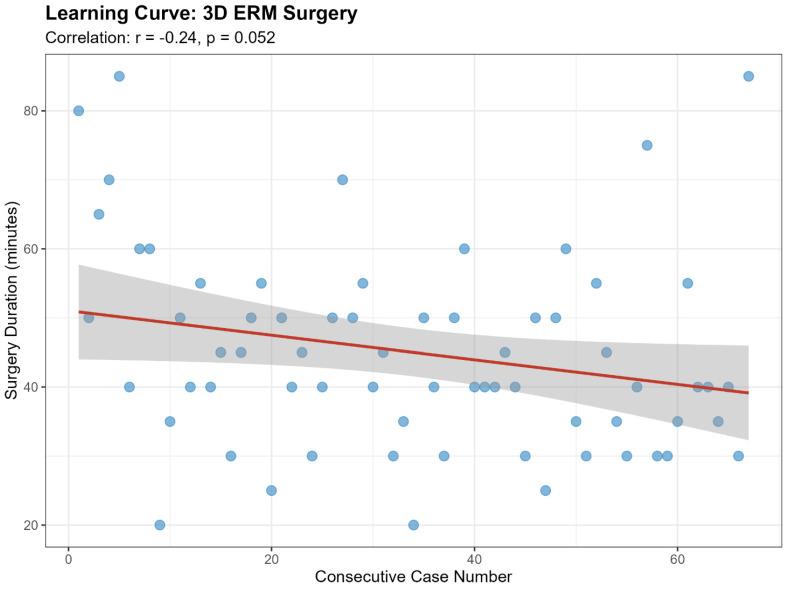
Scatter plot with linear regression line illustrating the learning curve for ERM surgery using the 3D heads-up visualization system. The graph displays surgical duration (minutes) over the consecutive case sequence (r = −0.24, *p* = 0.052). ERM = epiretinal membrane.

Analysis of the learning curve for the 3D group showed a weak, non-significant trend toward reduced surgical time over consecutive cases (Pearson’s r = −0.24), approaching statistical significance (*p* = 0.052) (Figure 1).

To assess the impact of the visualization system on surgical duration, a specific multivariate linear regression model was constructed for the ERM cohort, adjusting for disease severity (ERM stage), combined phacoemulsification, age, and baseline visual acuity.

As expected, combined cataract surgery significantly prolonged the operative time, by approximately 14.7 min (*p* < 0.001). Importantly, even after adjusting for the imbalance in preoperative staging (higher prevalence of stage 4 in the conventional group), the 3D heads-up system remained significantly faster. The model demonstrated an independent reduction in surgical duration of 5.5 min for the 3D group compared to the conventional microscope (coefficient: −5.49 [95% CI: −10.8 to −0.15], *p* = 0.044).

#### 3.2.2. Functional and Anatomical Outcomes

Visual acuity improved in both groups. At 6 months postoperatively, there was no significant difference in absolute BCVA between the conventional (0.20 logMAR) and 3D groups (0.20 logMAR) (*p* = 0.895). Regarding visual gain, the delta BCVA was statistically higher in the conventional group (median improvement 0.40 logMAR) compared to the 3D group (0.30 logMAR) (*p* = 0.048).

Anatomical success was achieved in both groups with significant CMT reduction. There were no significant differences in final CMT (*p* = 0.323) or delta CMT reduction (*p* = 0.063). The incidence of postoperative CME was low and comparable between groups (~20%, *p* = 1.000). Analysis of structural biomarkers revealed no significant differences in iatrogenic damage to the nerve fiber layer. The incidence of SANFL defects was similar at 1 month (48.9% vs. 45.3%, *p* = 0.841) and decreased over time in both groups. The appearance of DONFL increased progressively during follow-up, as expected, but showed no significant difference between the conventional and 3D visualization systems at 6 months (58.3% vs. 64.9%, *p* = 0.634). Results are summarized in Table 3.

### 3.3. MH

A total of 60 eyes (30 in the conventional microscope group and 30 in the 3D heads-up group) were analyzed. Age, gender, and lens status were well-balanced between the two groups (*p* > 0.05).

However, a significant difference was noted in the preoperative staging (*p* = 0.025): the conventional group had a higher proportion of stage 4 holes (83.3%) compared to the 3D group (53.3%). Despite this difference in staging, the median MLD of the holes was statistically comparable, with the 3D group presenting slightly larger holes (median: 372 µm vs. 323 µm; *p* = 0.124).

Baseline BCVA and CMT showed no significant differences (Table 4).

#### 3.3.1. Surgical Efficiency

**Figure 2:**
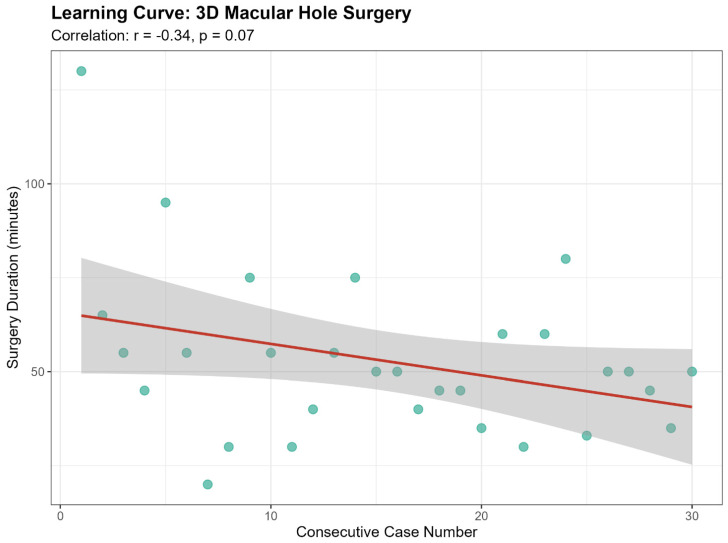
Scatterplot with linear regression line evaluating the learning curve for MH surgery using the 3D heads-up visualization system. The graph displays surgical duration (minutes) over the consecutive case sequence (r = −0.34, *p* = 0.07). MH = macular hole.

As for surgical duration the 3D heads-up group showed a trend toward shorter surgical times (median: 50.0 min) compared to the conventional group (median: 60.0 min), approaching statistical significance (*p* = 0.067). Consistent with the ERM findings, the intraoperative illumination intensity was significantly lower in the 3D group (median: 15 vs. 30; *p* < 0.001). The use of combined phacoemulsification (73.3% in both groups) and the inverted flap technique (36.7% vs. 30.0%, *p* = 0.785) was similar (Table 4). Analysis of the 3D group showed a weak negative correlation between case sequence and surgical duration (r = −0.34, *p* = 0.07), indicating no demonstrable or steep learning curve (Figure 2).

#### 3.3.2. Anatomical and Functional Outcomes

The primary anatomical success rate (type 1 closure) was high in both groups. The closure rate was 96.6% in the conventional group and 88.0% in the 3D group. This difference was not statistically significant (*p* = 0.326), indicating comparable efficacy between the two systems. Both groups achieved visual improvement. There were no statistically significant differences in BCVA at 1, 3, or 6 months. The median visual gain (delta BCVA) at 6 months was 0.35 logMAR in the conventional group and 0.20 logMAR in the 3D group (*p* = 0.504).

No significant differences were found in postoperative CMT or retinal thinning between the groups. Analysis of retinal nerve fiber layer integrity showed no significant advantage or disadvantage for the 3D system. The incidence of postoperative CME was identical (6.7% vs. 6.7%, *p* = 1.000). The appearance of SANFL defects at 1 month was slightly higher in the 3D group (42.9% vs. 27.3%), but not statistically significant (*p* = 0.374). Similarly, the prevalence of DONFL at 6 months was comparable (64.7% vs. 63.6%, *p* = 1.000) (Table 5).

### 3.4. RD

A total of 66 eyes were included (37 conventional, 29 3D). The 3D group was significantly older (66.6 vs. 59.4 years, *p* = 0.028), while the prevalence of “macula off” status was high and identical in both groups (~80%, *p* = 1.000).

Regarding surgical approach, the conventional group underwent combined PPV and scleral buckling (PPV + SB) significantly more often than the 3D group (75.7% vs. 41.4%, *p* = 0.006). Tamponade choice also differed significantly (*p* < 0.001), with more frequent use of SF6 in the 3D group (44.8%) and C3F8 in the conventional group (48.6%) (Table 6).

#### 3.4.1. Efficiency and Safety

**Figure 3:**
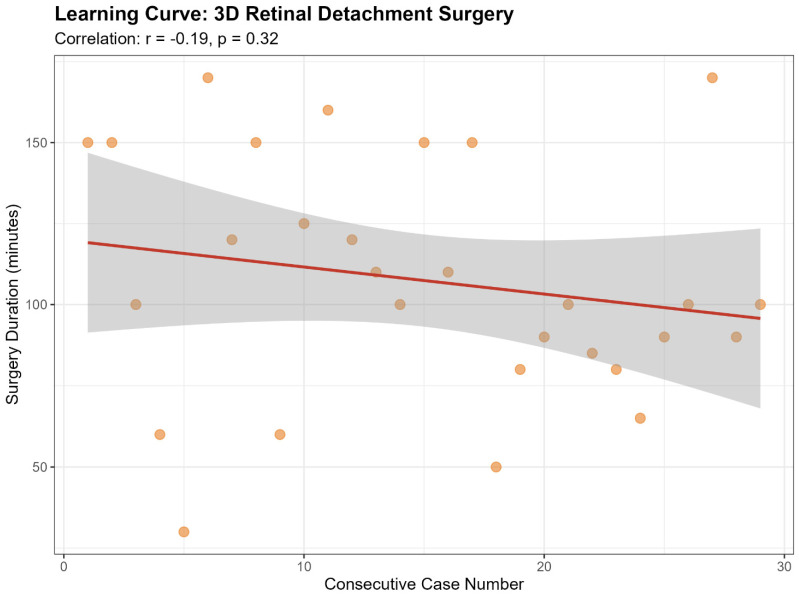
Scatterplot with linear regression line assessing the learning curve for RD surgery using the 3D heads-up visualization system. The graph displays surgical duration (minutes) over the consecutive case sequence (r = −0.19, *p* = 0.32). RD = retinal detachment.

3D surgery was performed with significantly lower illumination (15 vs. 30, *p* = 0.002). Surgical duration was shorter in the 3D group (100 min vs. 120 min), though this did not reach statistical significance (*p* = 0.128). No significant learning curve was observed for RD surgery (*p* = 0.32) (Figure 3).

#### 3.4.2. Anatomical and Functional Outcomes

The primary reattachment rate was high in both groups (94.6% conventional vs. 89.7% 3D, *p* = 0.647). RD recurrence within 3 months occurred in 8.1% of conventional cases and 13.8% of 3D cases (*p* = 0.690).

Final BCVA at 6 months was identical between groups (median 0.70 logMAR, *p* = 0.492). Although the conventional group showed a larger delta BCVA (0.90 vs. 0.30), the difference was not statistically significant (*p* = 0.160), likely due to the high variability of functional recovery in macula-off detachments (Table 7).

## 4. Discussion

The results of this study indicate that the use of 3D visualization systems in vitreoretinal surgery yielded outcomes comparable to the traditional operating microscope in terms of both anatomical success and functional outcomes across all major vitreoretinal conditions analyzed. When considering the overall population, the rates of MH closure, ERM removal, and retinal reattachment in RRD were largely comparable between the two visualization settings, consistent with the data reported in previous studies [5,7]. In our cohort, the median visual gain was significantly higher in the conventional group than in the 3D group. However, this finding is explained by the significant baseline imbalance in disease severity rather than visual acuity alone. The conventional cohort contained a significantly higher proportion of advanced macular pathologies (e.g., stage 4 ERMs), which structurally allow for a larger numerical functional rebound postsurgery. Ultimately, both visualization systems allowed patients to reach the same final visual potential, confirming that the digital platform does not compromise functional rehabilitation. Furthermore, the multivariate adjustment accounted for combined phacoemulsification. Since the rate of simultaneous cataract extraction was perfectly balanced between the conventional and 3D groups (~64%, *p* = 1.000), it did not bias the functional comparison.

The main advantage of the 3D group in the present study concerned surgical time, which was significantly shorter compared to conventional microscopy. Subgroup analysis revealed that this time reduction was particularly evident in ERM, whereas no significant difference was observed in cases of MH and RDD, likely due to the greater technical complexity and duration inherent to the procedures. Because all procedures were performed by the same highly experienced senior surgeon, this difference may not be attributable to varying surgical efficacy or experience. Rather, it reflects the specific technological benefits of the 3D system, as ERM peeling heavily relies on high-magnification macular micromanipulation.

From a technical perspective, we hypothesize that the reduced operating times observed in ERM may be attributable to the enhanced intraoperative visualization provided by the 3D system, including enhanced magnification, increased image sharpness, extended depth of field, stable stereoscopic perception, and better discrimination of retinal planes. The option to use custom color filters (such as the blu boost mode etc.) and the reduced need for visual accommodation by the surgeon likely contribute to greater precision and procedural confidence [12,13].

In our series, these technical benefits translated into a mean surgical time reduction of around 10 min per procedure, along with significantly lower endoillumination levels, confirming that optimal visualization can be achieved under safer light conditions. Similarly, other authors have reported that 3D digital visualization allows for surgery at lower machine endoillumination settings. While this theoretically may reduce the risk of retinal phototoxicity, it must be noted that our recorded values reflect system settings rather than direct intraocular radiometric measurements, leaving definitive claims on phototoxicity speculative at this stage. However, the subjective improvement in visual comfort for the surgical team remains a tangible benefit [14]. While univariate analysis suggested a reduction in operative time, the multivariate regression confirmed that this was not due to case selection. Even after adjusting for combined phacoemulsification, the 3D system saved an average of around 11 min per case (*p* = 0.011). This efficiency may be attributed to the enhanced depth of field, which reduces the need for continuous refocusing, and the ability to visualize the peripheral retina without excessive scleral depression. In our ERM cohort, univariate analysis initially suggested a 10 min reduction in surgical time with the 3D system. However, the conventional group presented with more advanced cases (stage 4). By applying a multivariate regression model that adjusted for disease severity, we isolated the true impact of the digital system. The analysis confirmed that the 3D system independently reduces surgical time by approximately 5.5 min per case (*p* = 0.044). This efficiency gain suggests that the enhanced depth of field and digital visualization facilitate the peeling maneuver itself, regardless of membrane complexity. Longitudinal analysis of surgical duration revealed only a weak trend rather than a demonstrable learning curve. Importantly, our temporal sensitivity analysis provides insights into the adoption of digital visualization. When restricting the comparison to the transitional overlap year, the surgical duration was comparable between the two systems. Since all procedures were performed by an experienced vitreoretinal surgeon, we suggest that this difference may reflect a specific technological learning curve. Transitioning to a heads-up display requires adaptation to new hand-eye coordination, altered depth perception, and room ergonomics. The global reduction in operative time observed in the overall cohort was largely driven by the later cases, indicating that once the initial technological adaptation phase is overcome, the 3D system may allow for optimized procedural efficiency. This is consistent with results observed by Mura et al. regarding improved surgical efficiency and shorter procedure times as operator experience with the 3D system increased [3].

In the context of RRD, the results should be interpreted with caution. In the conventional microscope group, a higher frequency of combined scleral buckling was observed, likely reflecting a greater baseline case complexity, which inherently prolongs surgical time and limits a direct comparison of visualization systems. Furthermore, a difference was noted regarding the choice of intraocular tamponade; however, since this decision is dictated by the anatomical and clinical configuration of the detachment rather than the visualization platform, it simply reflects the aforementioned heterogeneity and baseline imbalance of the two cohorts.

Overall, this study features several strengths, including a balanced group distribution, monocentric design, and the fact that all surgeries were performed by a single experienced vitreoretinal surgeon, reducing inter-operator variability and ensuring procedural uniformity. The detailed analysis of anatomical, functional, and temporal parameters, along with the assessment of the learning curve, further strengthens the methodological reliability of the findings. Nevertheless, some limitations should be acknowledged: the retrospective and non-randomized nature of the study and the heterogeneity of vitreoretinal diseases included. A primary limitation is inherent indication bias, as baseline imbalances suggest the surgeon preferentially selected the conventional microscope for more complex cases. Although multivariate regression was employed to adjust for these observed discrepancies, such statistical adjustment only mitigates, but cannot entirely eliminate residual confounding from unmeasured variables. Moreover, the overlap between learning effects and technological factors and the procedural disparities (use of scleral buckle, tamponade type) in RRD cases may have impacted the surgical timing. Finally, the endoillumination values were based on the surgical platform’s displayed settings rather than objective photometric or radiometric verification at the retinal plane.

In terms of implications, our results support the adoption of 3D visualization as a safe, efficient, and ergonomically advantageous technology, with clear benefits in standard macular procedures such as ERM and MH. Future developments may involve integrating 3D visualization with intraoperative optical coherence tomography (iOCT) and artificial intelligence (AI)–based tools for real-time identification of peeling planes, automated traction analysis, and procedural feedback [15,16]. Further prospective, randomized studies with stratification by pathology and technique, standardized criteria for tamponade and scleral buckling, and additional assessments of phototoxicity, ergonomics, and cost-effectiveness will be essential to confirm and extend these findings.

In conclusion, 3D vitreoretinal surgery demonstrated comparable anatomical and functional outcomes to conventional microscopy, while offering significant reductions in surgical time and improved intraoperative management through enhanced visualization and surgeon comfort. Although its role in more complex scenarios remains to be fully defined, the trajectory is clear: 3D visualization represents a mature, reliable technology that may serve as a valuable adjunct in modern vitreoretinal practice.

## 5. Conclusions

The consistency of our results and their alignment with the existing literature strengthen the validity of our observations. The 3D heads-up visualization system does not replace the traditional operating microscope, but it may help to overcome some of its limitations, offering a new surgical approach that is more reliable, efficient, and ergonomic, capable of providing an enhanced and shared view that can improve both the individual surgical experience and the collaboration within the operating team. In the future, integration of the 3D visualization system with advanced technologies such as iOCT and AI–based tools could pave the way for vitreoretinal surgery that is even safer, more personalized, and more predictive.

## Figures and Tables

**Table 1:** Baseline demographics, surgical indications, and global outcomes of the overall study cohort.

	Conventional Microscope	3D Heads-Up	*p*-Value
**n**	122	126	
**Age (median [IQR])**	70.03 [64.34, 77.80]	71.97 [64.76, 77.48]	0.555
**Gender = male (%)**	62 (50.8)	61 (48.4)	0.800
**Indication (%)**			0.356
**ERM**	55 (45.1)	67 (53.2)	
**MH**	30 (24.6)	30 (23.8)	
**RD**	37 (30.3)	29 (23.0)	
**Combined phaco = yes (%)**	79 (64.8)	81 (64.3)	1.000
**Surgery duration (median [IQR])**	60.00 [50.00, 90.00]	50.00 [40.00, 75.00]	**0.001**
**Preop BCVA (median [IQR])**	1.00 [0.50, 1.40]	0.70 [0.40, 1.00]	**0.001**
**BCVA 6M (median [IQR])**	0.40 [0.10, 0.70]	0.20 [0.10, 0.70]	0.120
**Delta BCVA 6M (median [IQR])**	0.40 [0.20, 0.90]	0.30 [0.10, 0.60]	**0.004**
**Postop CME**	26 (21.3)	22 (17.5)	0.521

**Abbreviations:** BCVA = best-corrected visual acuity; CME = cystoid macular edema; ERM = epiretinal membrane; IQR = interquartile range; MH = macular hole; RD = retinal detachment. Bold and background color for a better reading of the table and for highlighting the variables studied.

**Table 2:** Baseline clinical characteristics and intraoperative parameters for patients undergoing surgery for ERM.

	Conventional Microscope	3D Heads-Up	*p*-Value
**n**	55	67	
**Age (median [IQR])**	75.49 [67.83, 80.85]	75.17 [68.02, 77.75]	0.474
**Gender = male (%)**	30 (54.5)	29 (43.3)	0.275
**ERM stage (%)**			0.005
**1**	3 (5.5)	1 (1.5)	
**2**	6 (10.9)	9 (13.4)	
**3**	8 (14.5)	27 (40.3)	
**4**	38 (69.1)	30 (44.8)	
**Combined phaco = yes (%)**	35 (63.6)	44 (65.7)	0.851
**Preop BCVA (median [IQR])**	0.70 [0.40, 1.00]	0.50 [0.40, 0.70]	0.132
**Preop CMT (median [IQR])**	459.00 [380.25, 522.75]	429.00 [374.00, 489.00]	0.278
**Surgery duration (median [IQR])**	50.00 [40.00, 62.50]	40.00 [35.00, 50.00]	**0.012**
**Illumination intensity (median [IQR])**	35.00 [30.00, 35.00]	15.00 [15.00, 15.00]	**<0.001**

**Abbreviations:** BCVA = best-corrected visual acuity; CMT = central macular thickness; ERM = epiretinal membrane; IQR = interquartile range. Bold and background color for a better reading of the table and for highlighting the variables studied.

**Table 3:** Postoperative functional, anatomical, and structural outcomes at 1, 3, and 6 months for the ERM cohort.

	Conventional Microscope	3D Heads-Up	*p*
**n**	55	67	
**BCVA 1M (median [IQR])**	0.30 [0.10, 0.40]	0.20 [0.10, 0.40]	0.598
**BCVA 3M (median [IQR])**	0.20 [0.00, 0.40]	0.20 [0.10, 0.30]	0.713
**BCVA 6M (median [IQR])**	0.20 [0.00, 0.40]	0.20 [0.10, 0.30]	0.895
**Delta BCVA 6M (median [IQR])**	0.40 [0.21, 0.70]	0.30 [0.10, 0.50]	**0.048**
**Postop 6M CMT (median [IQR])**	376.00 [329.00, 414.00]	383.50 [340.25, 421.75]	0.323
**Delta CMT 6M (median [IQR])**	68.00 [23.00, 128.00]	42.00 [11.75, 98.00]	0.063
**Postop CME**	11 (20.0)	13 (19.4)	1.000
**SANFL 1M**	23 (48.9)	24 (45.3)	0.841
**SANFL 3M**	7 (25.0)	7 (19.4)	0.762
**SANFL 6M**	2 (5.7)	2 (5.4)	1.000
**DONFL 1M**	6 (12.8)	13 (24.5)	0.201
**DONFL 3M**	13 (46.4)	15 (41.7)	0.801
**DONFL 6M**	21 (58.3)	24 (64.9)	0.634

**Abbreviations:** BCVA = best-corrected visual acuity; CME = cystoid macular edema; CMT = central macular thickness; DONFL = dissociated optic nerve fiber layer; ERM = epiretinal membrane; IQR = interquartile range; SANFL = swelling of the arcuate nerve fiber layer. Bold and background color for a better reading of the table and for highlighting the variables studied.

**Table 4:** Baseline demographics, clinical staging, and intraoperative variables for the MH cohort.

	Conventional Microscope	3D Heads-Up	*p*
**n**	30	30	
**Age (median [IQR])**	70.23 [65.74, 77.45]	69.05 [64.31, 76.48]	0.425
**Gender = male (%)**	10 (33.3)	11 (36.7)	1.000
**MH stage = 4 (%)**	25 (83.3)	16 (53.3)	**0.025**
**MH size, microns (median [IQR])**	323.00 [234.00, 407.00]	372.00 [287.00, 562.50]	0.124
**Lens status (%)**			1.000
**0**	23 (76.7)	22 (73.3)	
**1**	7 (23.3)	7 (23.3)	
**2**	0 (0.0)	1 (3.3)	
**Preop BCVA (median [IQR])**	1.00 [0.55, 1.08]	0.70 [0.40, 1.00]	0.272
**Preop CMT (median [IQR])**	355.50 [285.00, 422.75]	322.50 [295.50, 378.50]	0.579
**Combined phaco**	22 (73.3)	22 (73.3)	1.000
**Inverted flap**	11 (36.7)	9 (30.0)	0.785
**Tamponade (%)**			0.214
**Air**	8 (26.7)	14 (46.7)	
**SF6**	19 (63.3)	12 (40.0)	
**C3F8**	3 (10.0)	4 (13.3)	
**Surgery duration (median [IQR])**	60.00 [50.00, 65.00]	50.00 [40.00, 58.75]	0.067
**Illumination intensity (median [IQR])**	30.00 [30.00, 35.00]	15.00 [15.00, 15.00]	**<0.001**

**Abbreviations:** BCVA = best-corrected visual acuity; C3F8 = perfluoropropane; CMT = central macular thickness; IQR = interquartile range; MH = macular hole; SF6 = sulfur hexafluoride. Bold and background color for a better reading of the table and for highlighting the variables studied.

**Table 5:** Postoperative functional, anatomical, and structural outcomes at 1, 3, and 6 months for the MH cohort.

	Conventional Microscope	3D Heads-Up	*p*-Value
**n**	30	30	
**MH closure = yes (%)**	28 (96.6)	22 (88.0)	0.326
**BCVA 1M (median [IQR])**	0.50 [0.30, 0.70]	0.45 [0.30, 0.70]	0.754
**BCVA 3M (median [IQR])**	0.50 [0.30, 0.92]	0.50 [0.20, 0.67]	0.398
**BCVA 6M (median [IQR])**	0.40 [0.20, 0.67]	0.45 [0.20, 0.70]	0.633
**Delta BCVA 6M (median [IQR])**	0.35 [0.12, 0.65]	0.20 [0.00, 0.73]	0.504
**Postop 6M CMT (median [IQR])**	287.50 [261.50, 319.50]	324.50 [278.00, 347.00]	0.181
**Delta CMT 6M (median [IQR])**	28.50 [12.00, 107.75]	28.50 [−29.50, 94.25]	0.352
**Postop CME**	2 (6.7)	2 (6.7)	1.000
**SANFL 1M**	6 (27.3)	12 (42.9)	0.374
**SANFL 3M**	0 (0.0)	1 (5.6)	1.000
**SANFL 6M**	0 (0.0)	2 (11.8)	0.193
**DONFL 1M**	2 (8.7)	4 (14.3)	0.678
**DONFL 3M**	7 (46.7)	8 (44.4)	1.000
**DONFL 6M**	14 (63.6)	11 (64.7)	1.000

**Abbreviations:** BCVA = best-corrected visual acuity; CME = cystoid macular edema; CMT = central macular thickness; DONFL = dissociated optic nerve fiber layer; MH = macular hole; IQR = interquartile range; SANFL = swelling of the arcuate nerve fiber layer. Bold and background color for a better reading of the table and for highlighting the variables studied.

**Table 6:** Baseline demographics, clinical characteristics, and intraoperative parameters for the RD cohort.

	Conventional Microscope	3D Heads-Up	*p*
**n**	37	29	
**Age (median [IQR])**	59.36 [54.14, 69.48]	66.57 [62.30, 75.27]	0.028
**Gender = male (%)**	22 (59.5)	21 (72.4)	0.309
**Lens Status = pseudophakic (%)**	14 (37.8)	14 (48.3)	0.457
**Macula status = macula off (%)**	29 (80.6)	23 (79.3)	1.000
**Preop BCVA (median [IQR])**	1.90 [1.17, 1.90]	1.00 [0.70, 2.70]	0.239
**PPV + scleral buckle (%)**	28 (75.7)	12 (41.4)	**0.006**
**Combined phaco = Yes (%)**	22 (59.5)	15 (51.7)	0.620
**Tamponade (%)**			**<0.001**
**Air**	4 (10.8)	0 (0.0)	
**SF6**	2 (5.4)	13 (44.8)	
**C3F8**	18 (48.6)	7 (24.1)	
**Silicon oil**	13 (35.1)	9 (31.0)	
**Surgery duration (median [IQR])**	120.00 [100.00, 150.00]	100.00 [85.00, 150.00]	0.128
**Illumination intensity (median [IQR])**	30.00 [30.00, 30.00]	15.00 [15.00, 15.00]	**0.002**

**Abbreviations:** BCVA = best-corrected visual acuity; C3F8 = perfluoropropane; IQR = interquartile range; PPV = pars plana vitrectomy; RD = retinal detachment; SF6 = sulfur hexafluoride. Bold and background color for a better reading of the table and for highlighting the variables studied.

**Table 7:** Postoperative anatomical and functional outcomes for the RD cohort.

	Conventional Microscope	3D Heads-Up	*p*-Value
**n**	37	29	
**RD reattachment = yes (%)**	35 (94.6)	26 (89.7)	0.647
**RD recurrence = yes (%)**	3 (8.1)	4 (13.8)	0.690
**BCVA 1M (median [IQR])**	1.00 [0.50, 1.00]	0.70 [0.30, 1.00]	0.360
**BCVA 3M (median [IQR])**	1.00 [0.50, 1.00]	0.70 [0.20, 1.00]	0.132
**BCVA 6M (median [IQR])**	0.70 [0.40, 1.00]	0.70 [0.20, 1.00]	0.492
**Delta BCVA 6M (median [IQR])**	0.90 [0.40, 1.20]	0.30 [0.10, 1.23]	0.160
**Postop CME = Yes (%)**	13 (35.1)	7 (24.1)	0.422

**Abbreviations:** BCVA = best-corrected visual acuity; CME = cystoid macular edema; IQR = interquartile range; RD = retinal detachment. Bold and background color for a better reading of the table and for highlighting the variables studied.

## Data Availability

The datasets generated and/or analyzed during the current study are not publicly available due to privacy and ethical restrictions regarding patient confidentiality, but are available from the corresponding author upon reasonable request.

## Abbreviations

The following abbreviations are used in this manuscript.

3D	three-dimensional
AI	artificial intelligence
BCVA	best-corrected visual acuity
C3F8	perfluoropropane
CME	cystoid macular edema
CMT	central macular thickness
DONFL	dissociated optic nerve fiber layer
ERM	epiretinal membrane
HDR	high dynamic range
ILM	internal limiting membrane
iOCT	intraoperative optical coherence tomography
IOL	intraocular lens
IQR	interquartile range
MH	macular hole
MLD	minimum linear diameter
PPV	pars plana vitrectomy
RD	retinal detachment
RRD	rhegmatogenous retinal detachment
SANFL	swelling of the arcuate nerve fiber layer
SB	scleral buckling
SD-OCT	spectral domain optical coherence tomography
SF6	sulfur hexafluoride
SO	silicone oil
